# Crystal structure of *N*,*N*′-bis[2-((benzyl){[5-(di­methyl­amino)naph­tha­len-1-yl]sulfonyl}amino)ethyl]naphthalene-1,8:4,5-tetracarboximide 1,2-di­chloro­benzene tris­olvate

**DOI:** 10.1107/S2056989016015188

**Published:** 2016-09-30

**Authors:** Miguel Ángel Claudio-Catalán, Felipe Medrano, Hugo Tlahuext, Carolina Godoy-Alcántar

**Affiliations:** a Centro de Investigaciones Químicas IICBA. Universidad Autónoma del Estado de, Morelos, Av. Universidad No. 1001, Col., Chamilpa, C. P. 62209, Cuernavaca Mor., México

**Keywords:** naphthalenedi­imide, dansyl amide, C—H⋯O, C—H⋯π and π–π inter­actions, crystal structure

## Abstract

The asymmetric unit of the title compound, C_56_H_50_N_6_O_8_S_2_·3C_6_H_4_Cl_2_, contains two half-mol­ecules of the parent, *A* and *B*, which both have crystallographic inversion symmetry, together with three 2,3-di­chloro­benzene mol­ecules of solvation. Mol­ecules *A* and *B* are conformationally similar, with dihedral angles between the central naphthalenedi­imide ring and the peripheral naphthalene and benzyl rings of 2.43 (7), 81.87 (7)° (*A*) and 3.95 (7), 84.88 (7)° (*B*), respectively. The conformations are stabilized by the presence of intra­molecular π–π inter­actions between the naphthalene ring and the six-membered di­imide ring of the central naphthalenedi­imide moiety, with ring centroid-to-centroid distances of 3.5795 (8) Å (*A*) and 3.5640 (8) Å (*B*). In the crystal, C—H⋯O hydrogen bonds link the mol­ecules into infinite supra­molecular chains along the *c* axis. These chains are inter­connected through C—H⋯π and offset π–π inter­actions, generating supra­molecular nanotubes which are filled by 1,2-di­chloro­benzene mol­ecules.

## Abstract 2

In the structure of the title compound, cooperative C—H⋯O=C, C—H⋯π and offset π–π inter­actions generate supra­molecular nanotubes which accommodate the 2,3-di­chloro­benzene solvent mol­ecules.

## Chemical context   

Non-covalent inter­actions concern a broad range of attractive effects with an equally varied energy contribution to bonding. An inter­esting group of inter­actions is one formed by the stabilizing weak polar contacts such C—H⋯ *X* (*X* = O, F, Cl, Br, I), C—H⋯π hydrogen bonds and offset π–π inter­actions. These inter­actions are involved in biological, materials, supra­molecular chemistry and crystal engineering (Desiraju, 1989 ▸; Desiraju & Steiner, 1999 ▸; Lehn, 1995 ▸; Steed & Atwood, 2000 ▸).

Naphthalimide is a highly fluorescent moiety that has been used as a construction block in the design of receptors and sensors that recognize charged species and other guests (Landey-Álvarez *et al.*, 2016 ▸). Aromatic imides show a highly efficient photo-induced electron transfer (PET) process that can be used as a signaling method in the building of sensors or on–off mol­ecular switches. In this sense, some researchers have proposed one approximation that involves the use of two different fluorescent units linked *via* a suitable spacer group characterized by PET or singlet–singlet energy transfer mechanisms (SSET) called dyads: such units are naphthalimide and dansyl amide. In a former study, these moieties were linked by methyl­ene units as a bridging group and only the photon-induced fluorescence switching was studied (Abad *et al.*, 2005 ▸). Later, inter­actions with different metallic ions were investigated (Shankar & Ramaiah, 2011 ▸). Actually, we have studied by single-crystal X-ray diffraction the mol­ecular structure of a naphthalimide-dansyl amide dyad and its inter­action in solution with anions and aromatic mol­ecules (Claudio-Catalán *et al.*, 2016 ▸). The ability of the dyad to function as a receptor of electron-rich guests and such aromatic compounds and anions are being studied by UV–Vis, fluorescence and NMR experiments. We have found that the dyad could inter­act with the guests tested through the aryl C—H⋯anion and aryl C—H⋯π inter­actions. In our ongoing research on naphthalimides as anion receptors, we report herein the synthesis and crystal structure of the title compound, a 1,2-di­chloro­benzene solvate, C_56_H_50_N_6_O_8_S_2_·3C_6_H_4_Cl_2_, (I), which has been shown to be inert to the presence of anions or neutral mol­ecules in solution probably due to high stability acquired by the overlap of the aromatic rings.
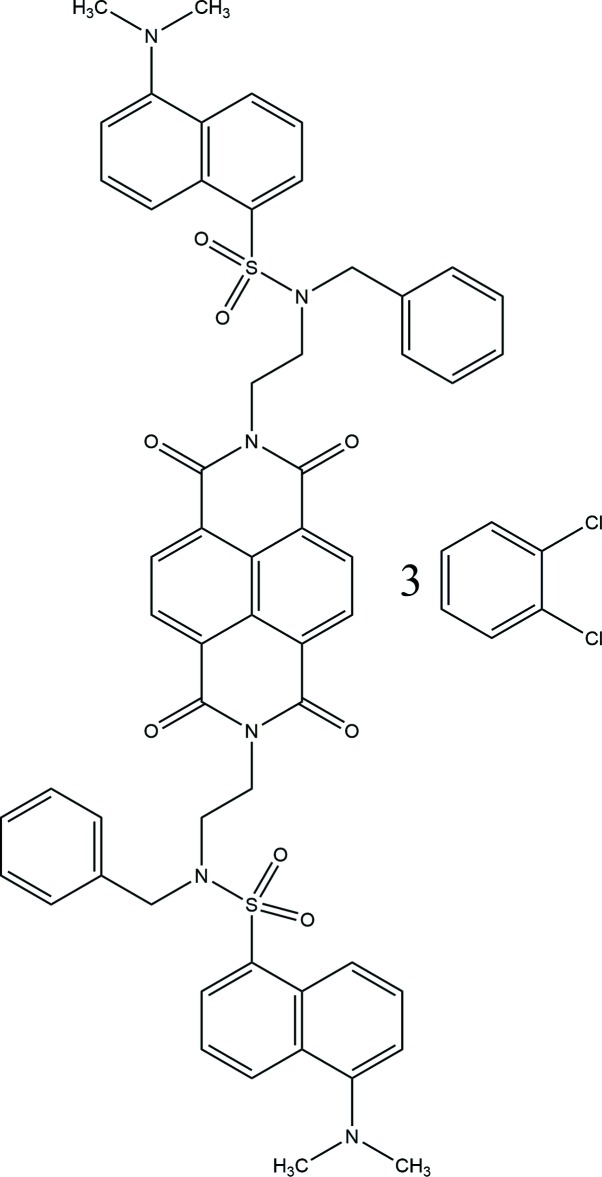


## Structural commentary   

**Figure 1:**
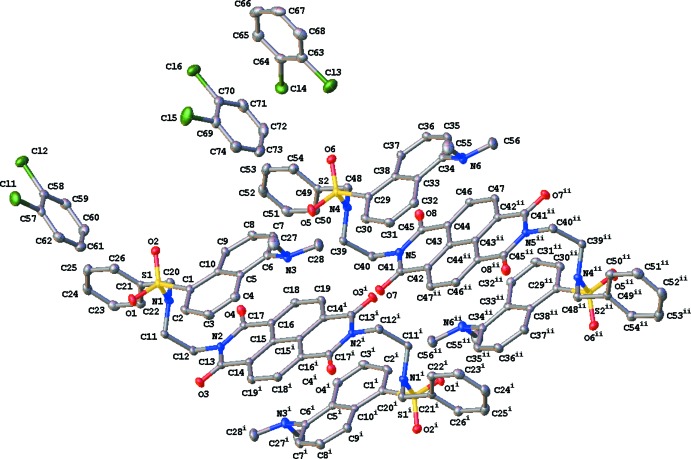
The mol­ecular structure of (I), showing the atom-labelling scheme. Displacement ellipsoids are drawn at the 50% probability level and H atoms are omitted for clarity. The inversion-related halves of mol­ecules *A* and *B* are generated by symmetry operations (i) −*x*, −*y* + 2, −*z* + 1 and (ii) −*x*, −*y* + 2, −*z* + 2, respectively.

**Figure 2:**
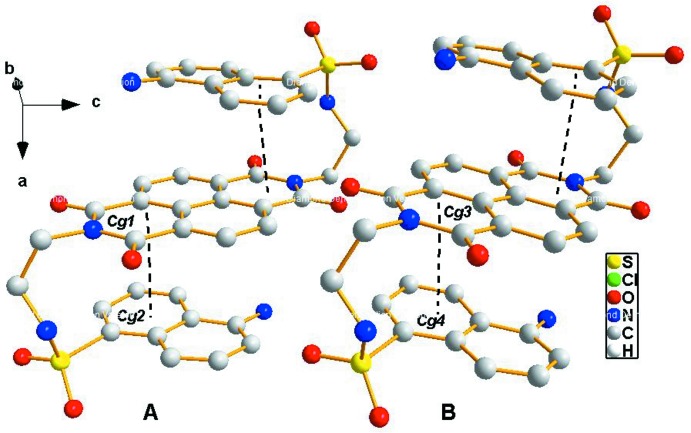
Mol­ecules *A* and *B* showing intra­molecular aromatic stacking. Dashed lines indicate the inter­actions between naphathalene­imide centroids *Cg*1 [C13–C17/N2 (*A*)] and *Cg*3 [C41–C45/N5 (*B*)] and aryl centroids *Cg*2 [C1–C5/C10 (*A*)] and *Cg*4 [C29–C33/C38 (*B*)]. Benzyl and methyl groups and H atoms are omitted.

The asymmetric unit of the title compound (I) contains two half-mol­ecules of the parent mol­ecule (*A* and *B*), both having crystallographic inversion symmetry [(i) −*x*, −*y* + 2, −*z* + 1 for (*A*) and (ii) −*x*, −*y* + 2, −*z* + 2 for (*B*)], and three 2,3-di­chloro­benzene mol­ecules of solvation (Fig. 1 ▸). The *N,N*-naphthalenedi­imide [N2/C13–C19 (*A*); N5/C41–C47 (*B*)] and aromatic dansyl groups [C1–C10 (*A*) and C29–C38 (*B*)] are almost planar with r.m.s. deviations of 0.0055, 0.0183, 0.0664 and 0.0698 Å, respectively. The two mol­ecules are conformationally similar with dihedral angles between the central naphthalenedi­imide ring and the peripheral naphthalene and benzyl rings, respectively, of 2.43 (7), 81.87 (7)° (*A*) and 3.95 (7), 84.88 (7)° (*B*). The conformations of *A* and *B* are stabilized by the presence of intra­molecular aromatic ring-stacking with distances of 3.5795 (8) and 3.5640 (8) Å for *Cg*1..*Cg*2 and *Cg*3⋯ *Cg*4, respectively [*Cg*1 and *Cg*3 are the centroids of naphathalene­imides C13–C17/N2 (*A*) and C41–C45/N5 (*B*) and *Cg*2 and *Cg*4 are the centroids of phenyl rings C1–C5/C10 (*A*) and C29–C33/C38 (*B*)] (Fig. 2 ▸).

## Supra­molecular features   

**Figure 3:**
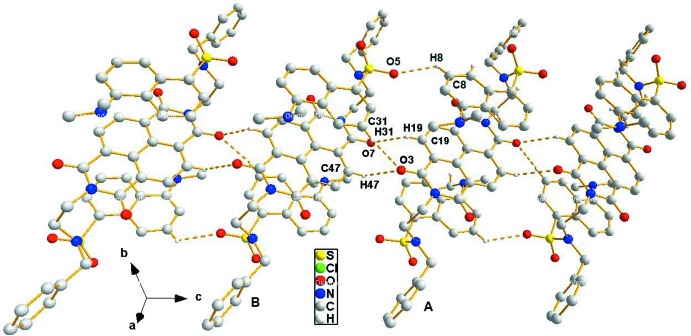
A view of the supra­molecular chain extending along the *c* axis, generated by C—H⋯O hydrogen bonds (dashed lines).

**Figure 4:**
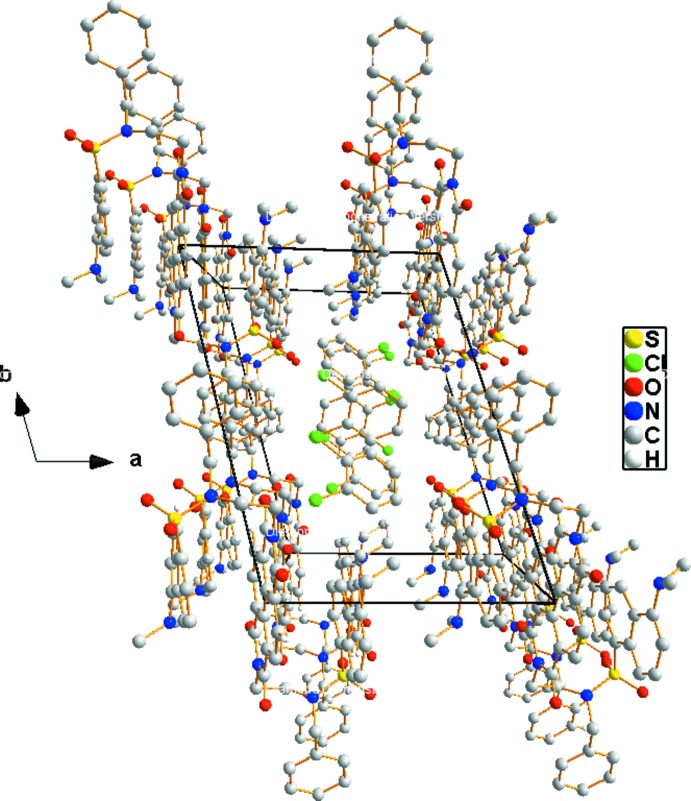
A perspective view along the *c* axis of the supra­molecular nanotube generated by cooperative C—H⋯π and offset π–π inter­actions, showing filling by 1,2-di­chloro­benzene mol­ecules.

**Figure 5:**
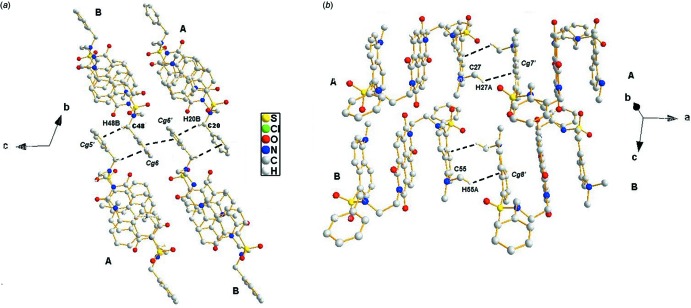
(*a*) A view of the C—H⋯π and offset π–π inter­actions between adjacent benzyl groups; (*b*) A view of additional C—H⋯π inter­actions between dansyl amide moieties. Hydrogen atoms not involved in the hydrogen-bonding inter­actions are omitted.

In the crystal, four C—H⋯O hydrogen bonds link the mol­ecules into infinite supra­molecular chains extending along the *c* axis (Fig. 3 ▸, Table 1 ▸). The chains are inter­connected through C—H⋯π and offset π–π inter­actions, generating channels which are filled by solvent mol­ecules (Fig. 4 ▸). The C—H⋯π inter­actions are between the benzyl groups with distances C48⋯*Cg*5′ = 3.6180 (17) and C20⋯*Cg*6′ = 3.6054 (17) Å (*Cg*5′ and *Cg*6′ are the centroids of the phenyl rings C21–C26 and C49–C54, respectively) (Fig. 5 ▸). The weak offset π–π inter­action is between adjacent phenyl rings with *Cg*6⋯*Cg*6′(−*x*, −*y* + 1, −*z* + 1) = 4.0277 (10) Å (*Cg*6 is the centroid of the C49–C54 phenyl ring). In addition, the dansyl groups show C—H⋯π inter­actions, with distances C27⋯*Cg*7′ = 3.585 (2) and C55⋯*Cg*8′ = 3.632 (2) Å (Fig. 6, Table 1 ▸) where *Cg*7′ and *Cg*8′ are the centroids of naphthyl ring systems C1–C10 and C29–C38, respectively. In the channel, the N⋯N distance is 12.5 Å. The solvent mol­ecules are inter­connected by C71—H71⋯*Cg*(C63–C68)] and C68—H68⋯Cl1 inter­actions and are also linked to the channel by C72—H72⋯O6 and C60—H60⋯O2 inter­actions (Table 1 ▸). In the crystal, there are also short Cl4⋯O4(−*x*, 1 − *y*, 1 − *z*) inter­actions [3.0923 (12) Å] and 22.6 Å^3^ solvent-accessible voids.

## Database survey   

A search of the Cambridge Structural Database (Version 5.37; Groom *et al.*, 2016 ▸) revealed the existence of 324 deposited naphthalenedi­imide structures. Amongst those, 94 structures are metal complexes. Supra­molecular constructs based on naphthalenedi­imide moieties with potential applications have been reported; for example PUNPAR (Wu *et al.*, 2015 ▸) and NUXJEX (Liu *et al.*, 2014 ▸) exhibit the formation of supra­molecular nanotubes through cooperative [C—H⋯O=C] inter­actions. In the same way, pseudorotaxanes BALVIU and GUNPEL (Colquhoun, *et al.*, 2010 ▸) and catenanes IVUNUI (Fallon, *et al.*, 2004 ▸), SUJZIG (Hamilton *et al.*, 1998 ▸) and WATYAR (Hansen *et al.*, 2000 ▸) have been prepared. Naphthalenedi­imides have also been used in mol­ecular recognition [HIRLAX (Schneebeli *et al.*, 2013 ▸), MUVJUJ (Shimizu, 2010 ▸), PUBPAE (Koshkakaryan *et al.*, 2009 ▸) and RULWUS (Ono *et al.*, 2015 ▸)].

## Synthesis and crystallization   

The title compound (I) was prepared from 2,7-bis­(2-benzyl­amino­eth­yl)naphthalenedi­imide (II), which was synthesized as follows. To a stirred solution of 1,4,5,8-naphthalene­tetra­carb­oxy­lic dianhydride (0.5 g, 1.86 mmol) in toluene (25 mL) was added *N*-benzyl­ethyl­endi­amine (0.56 mL, 0.56 g, 3.73 mmol) followed by the addition of tri­ethyl­amine (0.52 mL, 0.377 g, 3.73 mmol). The reaction mixture was heated to reflux with azeotropic removal of water using a Dean–Stark trap, for 24 h. The solution was cooled and the solvent was removed under reduced pressure. The resultant oil was purified by column chromatography on silica gel (CH_2_Cl_2_–MeOH 95:05). Compound (II) was obtained as a yellow solid (0.777 g, 78%). M.p. 482–484 K. IR (neat): 3314, 2817, 1700, 1655, 1579, 1454 cm^−1^. RMN ^1^H (400 MHz, CDCl_3_) δ: 1.59 (*s*, 2H, NH), 3.03 (*t*, *J* = 6.4 Hz, 4H, CH_2_NH), 3.84 (*s*, 4H, CH_2_Ph), 4.38 (*t*, *J* = 6.4 Hz, 4H, NCH_2_), 7.16–7.30 (*m*, 10H, H_aromatic_), 8.74 (*s*, 4H, H_aromatic_). RMN ^13^C (100 MHz, CDCl_3_) δ: 40.6 (2C, NCH_2_), 47.0 (2C, CH_2_NH), 53.7 (2C, CH_2_Ph), 126.8 (4C), 126.9 (2C), 127.1 (2C), 128.3 (4C), 128.5 (4C), 131.2 (4C), 140.4 (2C), 163.2 (C=O). MS (FAB^+^): *m*/*z* (%) 533 (37) [*M*]; HRMS (FAB^+^): calculated for C_32_H_29_O_4_N_4_ [*M*], *m*/*z* 533.2189; found for [*M*], *m*/*z* 533.2142.

Synthesis of *N*,*N*′-bis[2-((benzyl){[5-(di­methyl­amino)naphtha­len-1-yl]sulfonyl}amino)ethyl]naphthalene-1,8:4,5-tetracarboximide 1,2-di­chloro­benzene tris­olvate (I). A mixture of 2,7-bis­(2-benzyl­amino­eth­yl)naphthalenedi­imide (II) (0.5 g, 0.937 mmol), dansyl chloride (0.505 g, l.874 mmol) and K_2_CO_3_ (0.259 g, 1.874 mmol) in chloro­form/water (4:1) (10 mL) was stirred at room temperature for 20 h. The organic layer was extracted with di­chloro­methane (2 x 20 mL), dried over anhydrous Na_2_SO_4_, filtered and concentrated under reduced pressure. Further purification was performed by flash chromatography on silica gel (CH_2_Cl_2_–MeOH 95:5). After treatment with diethyl ether, the unsolvated title compound was obtained as a yellow solid (0.936 g, 100%). Crystallization from a chloro­form:1,2-di­chloro­benzene mixture afforded suitable crystals of the solvated compound (I) for the X-ray crystallographic analysis. M.p. 516–517 K. IR (neat): cm^−1^. RMN ^1^H (400 MHz, CDCl_3_) δ: 2.59 [*s*, 12H, (CH_3_)_2_N], 3.69 (*t*, *J* = 5.4 Hz, 4H, CH_2_NSO_2_), 4.28 (*t*, *J* = 5.4 Hz, 4H, CH_2_NCO), 4.85 (*s*, 4H, CH_2_Ph), 6.73 (*d*, *J* = 7.2 Hz, 2H, H_aromatic_), 7.18 (*dd*, *J* = 8.4, 7.2 Hz, 2H, H_aromatic_), 7.22–7.29 (*m*, 12H, H_aromatic_), 7.89 (*d*, *J* = 8.4 Hz, 2H, H_aromatic_), 7.97 (*t*, *J* = 8.8 Hz, 2H, H_aromatic_), 8.08 (*dd*, *J* = 7.2, 1.2 Hz, 2H, H_aromatic_), 8.42 (*s*, 4H, H_aromatic_). RMN ^13^C (100 MHz, CDCl_3_) δ: 37.5 (2C, CH_2_NCO), 43.1 (2C, CH_2_NSO_2_), 45.3 (4C, (CH_3_)_2_N), 49.9 (2C, CH_2_Ph), 114.4 (2C), 119.1 (2C), 123.0 (2C), 125.9 (4C),126.3 (2C), 128.1 (2C), 128.3 (2C), 128.9 (4C), 129.1 (4C), 129.2 (2C), 129.7 (2C), 130.1 (2C), 130.5 (2C), 130.6 (4C), 134.9 (2C), 135.6 (2C), 151.3 (2C), 162.9 (C=O). MS (FAB^+^): *m*/*z* (%) 999 (31) [*M* + H]^+^; HRMS (FAB^+^): calculated for C_21_H_19_O_2_N_2_ [*M* + H]^+^, *m*/*z* 999.3210; found for [*M* + H]^+^, *m*/*z* 999.3365. UV/Vis three bands CH_3_Cl: λ nm (∊, M^−1^ cm^−1^): 350 (24698), 362 (25362), 383 (29604).

## Refinement   

Crystal data, data collection and structure refinement details are summarized in Table 2. ▸ Aromatic, methyl­ene and methyl H atoms were positioned geometrically and were constrained using the riding-model approximation [C—H = 0.95–0.98 Å with *U*_iso_(H) = 1.5*U*_eq_(C-methyl) and 1.5*U*_eq_(C) for other H atoms].

## Supplementary Material

Crystal structure: contains datablock(s) I. DOI: 10.1107/S2056989016015188/zs2369sup1.cif

Structure factors: contains datablock(s) I. DOI: 10.1107/S2056989016015188/zs2369Isup2.hkl

CCDC reference: 1506790

Additional supporting information: crystallographic information; 3D view; checkCIF report

## Figures and Tables

**Table 1:** Hydrogen-bond geometry (Å, °) *Cg*, *Cg*5′, *Cg*6′, *Cg*7′ and *Cg*8′ are the centroids of atoms C63–C68, C21–C26, C49–C54, C1–C10 and C29–C38, respectively.

*D*—H⋯*A*	*D*—H	H⋯*A*	*D*⋯*A*	*D*—H⋯*A*
C8—H8⋯O5	0.95	2.53	3.1926 (19)	127
C19—H19⋯O7	0.95	2.55	3.2615 (19)	132
C31—H31⋯O3^i^	0.95	2.59	3.3818 (19)	141
C47—H47⋯O3^ii^	0.95	2.58	3.2148 (19)	125
C60—H60⋯O2	0.95	2.54	3.335 (2)	142
C68—H68⋯Cl1^ii^	0.95	2.79	3.5922 (19)	142
C72—H72⋯O6	0.95	2.50	3.293 (2)	141
C71—H71⋯*Cg*	0.95	2.99	3.813 (2)	145
C55—H55*A*⋯*Cg*8′^iii^	0.98	2.94	3.632 (2)	129
C27—H27*A*⋯*Cg*7′^iv^	0.98	3.03	3.585 (2)	117
C20—H20*B*⋯*Cg*6′^v^	0.99	3.12	3.6054 (17)	111
C48—H48*B*⋯*Cg*5′^v^	0.99	3.13	3.6180 (17)	112

Symmetry codes: (i) 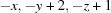; (ii) 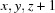; (iii) 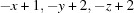; (iv) 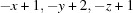; (v) 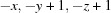.

**Table 2:** Experimental details

Crystal data	
Chemical formula	C_56_H_50_N_6_O_8_S_2_·3C_6_H_4_Cl_2_
*M*_r_	1440.11
Crystal system, space group	Triclinic, *P* 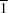
Temperature (K)	100
*a*, *b*, *c* (Å)	12.17737 (14), 17.2876 (2), 17.8916 (2)
α, β, γ (°)	110.9544 (12), 96.2760 (11), 103.5159 (10)
*V* (Å^3^)	3341.91 (8)
*Z*	2
Radiation type	Cu *K*α
μ (mm^−1^)	3.44
Crystal size (mm)	0.20 × 0.10 × 0.05

Data collection	
Diffractometer	Agilent SuperNova, Dual, Cu at zero, EosS2
Absorption correction	Multi-scan (*CrysAlis PRO*; Agilent, 2014 ▸)
*T*_min_, *T*_max_	0.874, 1.000
No. of measured, independent and observed [*I* > 2σ(*I*)] reflections	54279, 13086, 11914
*R*_int_	0.026
(sin θ/λ)_max_ (Å^−1^)	0.620

Refinement	
*R*[*F* ^2^ > 2σ(*F* ^2^)], *wR*(*F* ^2^), *S*	0.034, 0.097, 1.03
No. of reflections	13086
No. of parameters	869
H-atom treatment	H-atom parameters constrained
Δρ_max_, Δρ_min_ (e Å^−3^)	0.74, −0.45

Computer programs: *CrysAlis PRO* (Agilent, 2014 ▸), *SHELXS97* (Sheldrick, 2008 ▸), *SHELXL2014/7* (Sheldrick, 2015 ▸), *DIAMOND* (Brandenburg, 1999 ▸) and *OLEX2* (Dolomanov *et al.*, 2009 ▸).

## supplementary crystallographic information

### Crystal data

**Table:**

C_56_H_50_N_6_O_8_S_2_·3C_6_H_4_Cl_2_	*F*(000) = 1492
*M**_r_* = 1440.11	*D*_x_ = 1.431 Mg m^−^^3^
Triclinic, *P*1	Melting point = 516–517 K
*a* = 12.17737 (14) Å	Cu *K*α radiation, λ = 1.54184 Å
*b* = 17.2876 (2) Å	Cell parameters from 29431 reflections
*c* = 17.8916 (2) Å	θ = 2.9–72.6°
α = 110.9544 (12)°	µ = 3.44 mm^−^^1^
β = 96.2760 (11)°	*T* = 100 K
γ = 103.5159 (10)°	Prism, orange
*V* = 3341.91 (8) Å^3^	0.20 × 0.10 × 0.05 mm
*Z* = 2	

### Data collection

**Table:**

Agilent SuperNova, Dual, Cu at zero, EosS2 diffractometer	13086 independent reflections
Radiation source: sealed X-ray tube, SuperNova (Cu) X-ray Source	11914 reflections with *I* > 2σ(*I*)
Mirror monochromator	*R*_int_ = 0.026
Detector resolution: 8.0769 pixels mm^-1^	θ_max_ = 73.0°, θ_min_ = 2.7°
ω scans	*h* = −15→15
Absorption correction: multi-scan (CrysAlis PRO; Agilent, 2014)	*k* = −21→21
*T*_min_ = 0.874, *T*_max_ = 1.000	*l* = −21→22
54279 measured reflections	

### Refinement

**Table:**

Refinement on *F*^2^	Primary atom site location: structure-invariant direct methods
Least-squares matrix: full	Hydrogen site location: inferred from neighbouring sites
*R*[*F*^2^ > 2σ(*F*^2^)] = 0.034	H-atom parameters constrained
*wR*(*F*^2^) = 0.097	*w* = 1/[σ^2^(*F*_o_^2^) + (0.056*P*)^2^ + 1.406*P*] where *P* = (*F*_o_^2^ + 2*F*_c_^2^)/3
*S* = 1.03	(Δ/σ)_max_ = 0.001
13086 reflections	Δρ_max_ = 0.74 e Å^−^^3^
869 parameters	Δρ_min_ = −0.45 e Å^−^^3^
0 restraints	

### Special details

**Table:**

Geometry. All esds (except the esd in the dihedral angle between two l.s. planes) are estimated using the full covariance matrix. The cell esds are taken into account individually in the estimation of esds in distances, angles and torsion angles; correlations between esds in cell parameters are only used when they are defined by crystal symmetry. An approximate (isotropic) treatment of cell esds is used for estimating esds involving l.s. planes.
Refinement. 1. Fixed Uiso At 1.2 times of: All C(H) groups, All C(H,H) groups At 1.5 times of: All C(H,H,H) groups 2.a Secondary CH2 refined with riding coordinates: C12(H12A,H12B), C11(H11A,H11B), C20(H20A,H20B), C39(H39A,H39B), C48(H48A, H48B), C40(H40A,H40B) 2.b Aromatic/amide H refined with riding coordinates: C2(H2), C18(H18), C4(H4), C23(H23), C19(H19), C3(H3), C22(H22), C9(H9), C25(H25), C8(H8), C73(H73), C7(H7), C74(H74), C24(H24), C26(H26), C71(H71), C72(H72), C47(H47), C32(H32), C30(H30), C62(H62), C46(H46), C51(H51), C36(H36), C50(H50), C37(H37), C31(H31), C61(H61), C54(H54), C35(H35), C60(H60), C53(H53), C52(H52), C59(H59), C65(H65), C67(H67), C68(H68), C66(H66) 2.c Idealised Me refined as rotating group: C28(H28A,H28B,H28C), C27(H27A,H27B,H27C), C56(H56A,H56B,H56C), C55(H55A,H55B, H55C)

### Fractional atomic coordinates and isotropic or equivalent isotropic displacement parameters (Å^2^)

**Table:**

	*x*	*y*	*z*	*U*_iso_*/*U*_eq_	
S1	0.19073 (3)	0.75685 (2)	0.24683 (2)	0.01493 (8)	
Cl6	0.67288 (4)	0.57176 (3)	0.54497 (3)	0.03455 (10)	
Cl5	0.69686 (4)	0.73661 (3)	0.50338 (3)	0.04428 (13)	
O3	−0.08861 (9)	0.89558 (7)	0.24342 (6)	0.0210 (2)	
O1	0.19908 (9)	0.77111 (7)	0.17289 (6)	0.0212 (2)	
O4	−0.08934 (9)	0.72885 (6)	0.39083 (6)	0.0194 (2)	
O2	0.26566 (9)	0.71343 (7)	0.27162 (6)	0.0197 (2)	
N2	−0.08971 (10)	0.81240 (7)	0.31685 (7)	0.0149 (2)	
N1	0.05830 (10)	0.70359 (7)	0.24087 (7)	0.0152 (2)	
C13	−0.07211 (12)	0.89229 (9)	0.31046 (9)	0.0157 (3)	
C1	0.21767 (11)	0.86101 (9)	0.32637 (9)	0.0148 (3)	
C2	0.22425 (12)	0.92925 (9)	0.30290 (9)	0.0185 (3)	
H2	0.2112	0.9190	0.2464	0.022*	
N3	0.35385 (11)	1.06595 (8)	0.61154 (8)	0.0203 (3)	
C15	−0.01802 (11)	0.96231 (9)	0.46334 (8)	0.0133 (3)	
C18	−0.01783 (12)	0.87352 (9)	0.54171 (9)	0.0170 (3)	
H18	−0.0290	0.8181	0.5437	0.020*	
C4	0.27689 (12)	1.02957 (9)	0.44374 (9)	0.0181 (3)	
H4	0.2967	1.0873	0.4834	0.022*	
C16	−0.03523 (11)	0.88034 (9)	0.46729 (9)	0.0139 (3)	
C23	−0.14700 (13)	0.41817 (10)	0.06594 (10)	0.0226 (3)	
H23	−0.2258	0.3848	0.0445	0.027*	
C17	−0.07277 (11)	0.80072 (9)	0.39066 (9)	0.0148 (3)	
C19	0.01643 (12)	0.94848 (9)	0.61479 (9)	0.0177 (3)	
H19	0.0270	0.9435	0.6661	0.021*	
C12	−0.13094 (12)	0.73496 (9)	0.23968 (9)	0.0169 (3)	
H12A	−0.1931	0.7419	0.2043	0.020*	
H12B	−0.1642	0.6838	0.2517	0.020*	
C5	0.27561 (11)	0.96116 (9)	0.47012 (9)	0.0148 (3)	
C10	0.23834 (11)	0.87377 (9)	0.41091 (9)	0.0140 (3)	
C21	−0.00149 (12)	0.54144 (9)	0.17070 (9)	0.0157 (3)	
C14	−0.03475 (12)	0.97082 (9)	0.38777 (9)	0.0150 (3)	
C3	0.25032 (13)	1.01428 (9)	0.36250 (10)	0.0202 (3)	
H3	0.2495	1.0608	0.3463	0.024*	
C6	0.30788 (11)	0.97820 (9)	0.55544 (9)	0.0166 (3)	
C22	−0.11631 (13)	0.49109 (9)	0.13944 (9)	0.0186 (3)	
H22	−0.1741	0.5065	0.1683	0.022*	
C9	0.22245 (12)	0.80530 (9)	0.43852 (9)	0.0168 (3)	
H9	0.1960	0.7468	0.4000	0.020*	
C11	−0.03476 (12)	0.71921 (9)	0.19387 (8)	0.0155 (3)	
H11A	−0.0676	0.6685	0.1412	0.019*	
H11B	−0.0020	0.7702	0.1815	0.019*	
C25	0.05176 (14)	0.44374 (10)	0.05536 (10)	0.0241 (3)	
H25	0.1097	0.4273	0.0272	0.029*	
C8	0.24521 (13)	0.82367 (10)	0.52047 (9)	0.0202 (3)	
H8	0.2306	0.7774	0.5384	0.024*	
C73	0.43748 (14)	0.73761 (12)	0.62140 (11)	0.0321 (4)	
H73	0.3867	0.7718	0.6371	0.039*	
C7	0.28998 (13)	0.90977 (10)	0.57924 (9)	0.0201 (3)	
H7	0.3079	0.9205	0.6357	0.024*	
C74	0.51894 (16)	0.75901 (11)	0.57842 (11)	0.0300 (4)	
H74	0.5243	0.8078	0.5647	0.036*	
C69	0.59299 (14)	0.70853 (10)	0.55541 (10)	0.0243 (3)	
C20	0.03168 (12)	0.62007 (9)	0.25087 (9)	0.0163 (3)	
H20A	−0.0326	0.6166	0.2802	0.020*	
H20B	0.1002	0.6189	0.2853	0.020*	
C70	0.58373 (13)	0.63686 (10)	0.57470 (10)	0.0226 (3)	
C24	−0.06324 (14)	0.39416 (10)	0.02411 (10)	0.0234 (3)	
H24	−0.0842	0.3441	−0.0256	0.028*	
C28	0.35179 (15)	1.08222 (11)	0.69698 (10)	0.0276 (3)	
H28A	0.2750	1.0524	0.7009	0.041*	
H28B	0.3692	1.1447	0.7286	0.041*	
H28C	0.4098	1.0606	0.7189	0.041*	
C26	0.08184 (13)	0.51720 (9)	0.12751 (9)	0.0199 (3)	
H26	0.1603	0.5515	0.1478	0.024*	
C71	0.50229 (16)	0.61617 (12)	0.61816 (11)	0.0308 (4)	
H71	0.4965	0.5673	0.6318	0.037*	
C27	0.47087 (14)	1.10772 (12)	0.60510 (11)	0.0308 (4)	
H27A	0.5270	1.0855	0.6283	0.046*	
H27B	0.4904	1.1706	0.6354	0.046*	
H27C	0.4729	1.0948	0.5474	0.046*	
C72	0.42945 (15)	0.66686 (13)	0.64163 (11)	0.0346 (4)	
H72	0.3738	0.6530	0.6717	0.042*	
S2	0.17486 (3)	0.76401 (2)	0.74465 (2)	0.01531 (8)	
Cl1	0.74045 (3)	0.73768 (3)	0.03177 (3)	0.03251 (10)	
Cl2	0.67800 (3)	0.56374 (2)	0.06086 (3)	0.03107 (10)	
O7	−0.11632 (9)	0.89353 (7)	0.74506 (6)	0.0215 (2)	
O5	0.17643 (9)	0.78095 (7)	0.67160 (6)	0.0225 (2)	
O8	−0.10021 (9)	0.72829 (6)	0.89241 (6)	0.0199 (2)	
O6	0.25383 (9)	0.72127 (7)	0.76537 (7)	0.0212 (2)	
N5	−0.10660 (10)	0.81110 (8)	0.81829 (7)	0.0157 (2)	
N4	0.04457 (10)	0.70822 (7)	0.74075 (7)	0.0154 (2)	
C47	0.03877 (12)	0.94984 (9)	1.11372 (9)	0.0171 (3)	
H47	0.0585	0.9454	1.1646	0.020*	
C41	−0.09349 (12)	0.89094 (9)	0.81190 (9)	0.0163 (3)	
C38	0.23320 (11)	0.87835 (9)	0.91056 (9)	0.0141 (3)	
C44	−0.03418 (11)	0.88033 (9)	0.96747 (9)	0.0144 (3)	
C49	−0.00293 (12)	0.54617 (9)	0.67090 (9)	0.0161 (3)	
C32	0.26568 (12)	1.03448 (9)	0.94680 (9)	0.0175 (3)	
H32	0.2866	1.0918	0.9874	0.021*	
C29	0.20294 (11)	0.86685 (9)	0.82676 (9)	0.0149 (3)	
N6	0.36162 (11)	1.06867 (8)	1.11139 (8)	0.0201 (3)	
C30	0.20231 (12)	0.93567 (9)	0.80535 (9)	0.0184 (3)	
H30	0.1833	0.9263	0.7493	0.022*	
C43	−0.02205 (11)	0.96206 (9)	0.96352 (8)	0.0136 (3)	
C57	0.61803 (13)	0.70800 (10)	0.06827 (9)	0.0209 (3)	
C62	0.54740 (14)	0.76180 (10)	0.08531 (10)	0.0250 (3)	
H62	0.5645	0.8131	0.0750	0.030*	
C46	−0.00313 (12)	0.87448 (9)	1.04134 (9)	0.0161 (3)	
H46	−0.0101	0.8194	1.0432	0.019*	
C45	−0.08202 (11)	0.80018 (9)	0.89182 (9)	0.0151 (3)	
C42	−0.05138 (12)	0.96965 (9)	0.88864 (9)	0.0153 (3)	
C39	−0.05221 (12)	0.72071 (9)	0.69431 (9)	0.0161 (3)	
H39A	−0.0843	0.6695	0.6419	0.019*	
H39B	−0.0233	0.7719	0.6814	0.019*	
C51	−0.14298 (14)	0.42729 (10)	0.55784 (10)	0.0250 (3)	
H51	−0.2212	0.3968	0.5306	0.030*	
C48	0.02496 (12)	0.62465 (9)	0.75062 (9)	0.0169 (3)	
H48A	−0.0395	0.6180	0.7793	0.020*	
H48B	0.0951	0.6266	0.7858	0.020*	
C36	0.25694 (13)	0.82636 (9)	1.01796 (9)	0.0196 (3)	
H36	0.2471	0.7796	1.0351	0.024*	
C40	−0.14825 (12)	0.73367 (9)	0.74141 (9)	0.0178 (3)	
H40A	−0.2126	0.7394	0.7069	0.021*	
H40B	−0.1783	0.6821	0.7536	0.021*	
C50	−0.11737 (13)	0.50007 (9)	0.63098 (10)	0.0202 (3)	
H50	−0.1784	0.5184	0.6538	0.024*	
C34	0.31473 (11)	0.98141 (9)	1.05462 (9)	0.0163 (3)	
C37	0.22504 (12)	0.80914 (9)	0.93661 (9)	0.0168 (3)	
H37	0.1974	0.7509	0.8976	0.020*	
C58	0.59165 (13)	0.63204 (10)	0.08181 (9)	0.0203 (3)	
C31	0.22989 (12)	1.02016 (9)	0.86658 (9)	0.0191 (3)	
H31	0.2236	1.0670	0.8521	0.023*	
C61	0.45182 (14)	0.74021 (11)	0.11743 (10)	0.0273 (3)	
H61	0.4038	0.7772	0.1298	0.033*	
C54	0.08498 (13)	0.51758 (9)	0.63686 (10)	0.0209 (3)	
H54	0.1633	0.5485	0.6633	0.025*	
C33	0.27227 (11)	0.96537 (9)	0.97068 (9)	0.0148 (3)	
C56	0.37005 (14)	1.08286 (11)	1.19736 (10)	0.0267 (3)	
H56A	0.2963	1.0521	1.2048	0.040*	
H56B	0.3882	1.1450	1.2306	0.040*	
H56C	0.4314	1.0610	1.2146	0.040*	
C35	0.30409 (13)	0.91200 (10)	1.07700 (9)	0.0194 (3)	
H35	0.3288	0.9219	1.1328	0.023*	
C60	0.42596 (14)	0.66501 (11)	0.13160 (11)	0.0275 (3)	
H60	0.3605	0.6507	0.1539	0.033*	
C53	0.05946 (15)	0.44438 (10)	0.56466 (10)	0.0251 (3)	
H53	0.1202	0.4250	0.5425	0.030*	
C52	−0.05465 (15)	0.39939 (10)	0.52484 (10)	0.0257 (3)	
H52	−0.0721	0.3496	0.4751	0.031*	
C59	0.49533 (14)	0.61065 (10)	0.11340 (10)	0.0243 (3)	
H59	0.4770	0.5587	0.1225	0.029*	
C55	0.47316 (14)	1.11330 (12)	1.09971 (11)	0.0323 (4)	
H55A	0.5340	1.0921	1.1187	0.048*	
H55B	0.4914	1.1758	1.1312	0.048*	
H55C	0.4682	1.1017	1.0415	0.048*	
Cl4	0.30187 (3)	0.43343 (3)	0.69251 (3)	0.03212 (10)	
Cl3	0.40852 (4)	0.62180 (3)	0.83265 (3)	0.03876 (11)	
C63	0.49571 (14)	0.55818 (10)	0.79349 (10)	0.0242 (3)	
C65	0.52000 (14)	0.42501 (11)	0.70186 (11)	0.0269 (3)	
H65	0.4878	0.3685	0.6599	0.032*	
C64	0.44887 (13)	0.47549 (10)	0.73244 (10)	0.0226 (3)	
C67	0.68559 (14)	0.54154 (12)	0.79409 (12)	0.0313 (4)	
H67	0.7667	0.5641	0.8151	0.038*	
C68	0.61463 (15)	0.59036 (11)	0.82303 (11)	0.0302 (4)	
H68	0.6469	0.6473	0.8641	0.036*	
C66	0.63803 (15)	0.45730 (12)	0.73272 (12)	0.0322 (4)	
H66	0.6870	0.4227	0.7126	0.039*	

### Atomic displacement parameters (Å^2^)

**Table:**

	*U*^11^	*U*^22^	*U*^33^	*U*^12^	*U*^13^	*U*^23^
S1	0.01687 (16)	0.01485 (15)	0.01071 (16)	0.00375 (12)	0.00225 (12)	0.00316 (13)
Cl6	0.0310 (2)	0.0289 (2)	0.0372 (2)	0.01418 (16)	0.00131 (17)	0.00403 (18)
Cl5	0.0503 (3)	0.0319 (2)	0.0516 (3)	0.00476 (19)	0.0321 (2)	0.0162 (2)
O3	0.0307 (6)	0.0202 (5)	0.0120 (5)	0.0091 (4)	0.0009 (4)	0.0061 (4)
O1	0.0241 (5)	0.0239 (5)	0.0123 (5)	0.0032 (4)	0.0045 (4)	0.0057 (4)
O4	0.0258 (5)	0.0134 (5)	0.0168 (5)	0.0037 (4)	0.0036 (4)	0.0050 (4)
O2	0.0198 (5)	0.0201 (5)	0.0182 (5)	0.0089 (4)	0.0034 (4)	0.0046 (4)
N2	0.0169 (5)	0.0141 (5)	0.0111 (6)	0.0045 (4)	0.0008 (4)	0.0026 (5)
N1	0.0160 (5)	0.0129 (5)	0.0151 (6)	0.0031 (4)	0.0000 (4)	0.0053 (5)
C13	0.0171 (6)	0.0164 (7)	0.0126 (7)	0.0062 (5)	0.0016 (5)	0.0043 (6)
C1	0.0128 (6)	0.0142 (6)	0.0134 (7)	0.0019 (5)	0.0005 (5)	0.0030 (5)
C2	0.0194 (7)	0.0205 (7)	0.0145 (7)	0.0039 (5)	0.0002 (5)	0.0079 (6)
N3	0.0178 (6)	0.0194 (6)	0.0144 (6)	0.0007 (5)	0.0001 (5)	0.0001 (5)
C15	0.0117 (6)	0.0154 (6)	0.0132 (7)	0.0053 (5)	0.0029 (5)	0.0053 (6)
C18	0.0202 (7)	0.0145 (6)	0.0178 (7)	0.0059 (5)	0.0042 (5)	0.0077 (6)
C4	0.0170 (6)	0.0138 (6)	0.0199 (7)	0.0023 (5)	0.0014 (5)	0.0048 (6)
C16	0.0127 (6)	0.0150 (6)	0.0136 (7)	0.0050 (5)	0.0027 (5)	0.0047 (5)
C23	0.0232 (7)	0.0163 (7)	0.0226 (8)	0.0006 (6)	−0.0023 (6)	0.0066 (6)
C17	0.0139 (6)	0.0166 (7)	0.0136 (7)	0.0049 (5)	0.0025 (5)	0.0055 (6)
C19	0.0229 (7)	0.0189 (7)	0.0125 (7)	0.0070 (6)	0.0029 (5)	0.0073 (6)
C12	0.0188 (7)	0.0148 (6)	0.0123 (7)	0.0036 (5)	−0.0007 (5)	0.0016 (5)
C5	0.0113 (6)	0.0165 (6)	0.0147 (7)	0.0033 (5)	0.0022 (5)	0.0046 (6)
C10	0.0118 (6)	0.0156 (6)	0.0138 (7)	0.0044 (5)	0.0020 (5)	0.0050 (5)
C21	0.0211 (7)	0.0130 (6)	0.0136 (7)	0.0058 (5)	0.0022 (5)	0.0060 (5)
C14	0.0151 (6)	0.0161 (6)	0.0139 (7)	0.0063 (5)	0.0022 (5)	0.0051 (6)
C3	0.0224 (7)	0.0156 (7)	0.0225 (8)	0.0038 (5)	0.0009 (6)	0.0096 (6)
C6	0.0122 (6)	0.0192 (7)	0.0139 (7)	0.0031 (5)	0.0013 (5)	0.0028 (6)
C22	0.0201 (7)	0.0163 (6)	0.0195 (7)	0.0057 (5)	0.0034 (6)	0.0070 (6)
C9	0.0191 (7)	0.0150 (6)	0.0139 (7)	0.0036 (5)	0.0018 (5)	0.0044 (6)
C11	0.0192 (6)	0.0144 (6)	0.0111 (6)	0.0046 (5)	−0.0002 (5)	0.0041 (5)
C25	0.0292 (8)	0.0214 (7)	0.0215 (8)	0.0097 (6)	0.0094 (6)	0.0058 (6)
C8	0.0245 (7)	0.0202 (7)	0.0179 (7)	0.0057 (6)	0.0041 (6)	0.0104 (6)
C73	0.0235 (8)	0.0393 (10)	0.0249 (9)	0.0132 (7)	0.0023 (7)	0.0013 (8)
C7	0.0220 (7)	0.0249 (7)	0.0119 (7)	0.0066 (6)	0.0020 (5)	0.0064 (6)
C74	0.0367 (9)	0.0268 (8)	0.0255 (9)	0.0121 (7)	0.0035 (7)	0.0084 (7)
C69	0.0245 (7)	0.0234 (7)	0.0206 (8)	0.0029 (6)	0.0062 (6)	0.0058 (6)
C20	0.0217 (7)	0.0132 (6)	0.0133 (7)	0.0049 (5)	0.0026 (5)	0.0049 (6)
C70	0.0207 (7)	0.0224 (7)	0.0187 (8)	0.0052 (6)	−0.0005 (6)	0.0033 (6)
C24	0.0339 (8)	0.0147 (7)	0.0160 (7)	0.0048 (6)	0.0017 (6)	0.0021 (6)
C28	0.0292 (8)	0.0279 (8)	0.0150 (8)	0.0050 (7)	0.0011 (6)	−0.0006 (6)
C26	0.0205 (7)	0.0174 (7)	0.0197 (7)	0.0048 (6)	0.0033 (6)	0.0056 (6)
C71	0.0371 (9)	0.0308 (9)	0.0239 (9)	0.0053 (7)	0.0069 (7)	0.0131 (7)
C27	0.0218 (8)	0.0295 (8)	0.0254 (9)	−0.0051 (6)	0.0004 (7)	0.0023 (7)
C72	0.0275 (8)	0.0441 (10)	0.0253 (9)	0.0039 (8)	0.0111 (7)	0.0085 (8)
S2	0.01726 (16)	0.01544 (16)	0.01142 (16)	0.00420 (12)	0.00325 (12)	0.00358 (13)
Cl1	0.02762 (19)	0.0374 (2)	0.0291 (2)	0.00037 (16)	0.01108 (16)	0.01363 (18)
Cl2	0.02618 (19)	0.02378 (19)	0.0357 (2)	0.01054 (15)	0.00128 (16)	0.00270 (17)
O7	0.0302 (6)	0.0203 (5)	0.0139 (5)	0.0094 (4)	0.0009 (4)	0.0064 (4)
O5	0.0268 (5)	0.0241 (5)	0.0130 (5)	0.0027 (4)	0.0052 (4)	0.0060 (4)
O8	0.0265 (5)	0.0138 (5)	0.0184 (5)	0.0043 (4)	0.0055 (4)	0.0062 (4)
O6	0.0199 (5)	0.0214 (5)	0.0211 (5)	0.0095 (4)	0.0042 (4)	0.0050 (4)
N5	0.0172 (5)	0.0146 (5)	0.0140 (6)	0.0051 (4)	0.0020 (4)	0.0043 (5)
N4	0.0172 (6)	0.0133 (5)	0.0147 (6)	0.0039 (4)	0.0009 (5)	0.0055 (5)
C47	0.0200 (7)	0.0192 (7)	0.0141 (7)	0.0073 (5)	0.0032 (5)	0.0081 (6)
C41	0.0170 (6)	0.0169 (7)	0.0154 (7)	0.0066 (5)	0.0027 (5)	0.0060 (6)
C38	0.0119 (6)	0.0157 (6)	0.0138 (7)	0.0037 (5)	0.0022 (5)	0.0052 (5)
C44	0.0132 (6)	0.0153 (6)	0.0155 (7)	0.0055 (5)	0.0039 (5)	0.0060 (6)
C49	0.0216 (7)	0.0132 (6)	0.0141 (7)	0.0049 (5)	0.0024 (5)	0.0065 (6)
C32	0.0168 (6)	0.0136 (6)	0.0197 (7)	0.0026 (5)	0.0031 (5)	0.0053 (6)
C29	0.0140 (6)	0.0141 (6)	0.0144 (7)	0.0030 (5)	0.0024 (5)	0.0040 (5)
N6	0.0185 (6)	0.0192 (6)	0.0152 (6)	0.0015 (5)	0.0011 (5)	0.0017 (5)
C30	0.0191 (7)	0.0205 (7)	0.0159 (7)	0.0045 (5)	0.0016 (5)	0.0090 (6)
C43	0.0123 (6)	0.0153 (6)	0.0145 (7)	0.0056 (5)	0.0040 (5)	0.0060 (6)
C57	0.0193 (7)	0.0250 (7)	0.0135 (7)	0.0009 (6)	0.0015 (6)	0.0060 (6)
C62	0.0282 (8)	0.0222 (7)	0.0227 (8)	0.0051 (6)	−0.0008 (6)	0.0099 (6)
C46	0.0187 (6)	0.0145 (6)	0.0175 (7)	0.0061 (5)	0.0050 (5)	0.0080 (6)
C45	0.0144 (6)	0.0167 (7)	0.0159 (7)	0.0059 (5)	0.0054 (5)	0.0070 (6)
C42	0.0153 (6)	0.0165 (7)	0.0151 (7)	0.0057 (5)	0.0039 (5)	0.0065 (6)
C39	0.0190 (7)	0.0148 (6)	0.0120 (7)	0.0043 (5)	0.0002 (5)	0.0039 (5)
C51	0.0270 (8)	0.0178 (7)	0.0230 (8)	0.0012 (6)	−0.0033 (6)	0.0059 (6)
C48	0.0220 (7)	0.0143 (6)	0.0145 (7)	0.0053 (5)	0.0030 (5)	0.0061 (6)
C36	0.0235 (7)	0.0191 (7)	0.0177 (7)	0.0066 (6)	0.0027 (6)	0.0093 (6)
C40	0.0188 (7)	0.0158 (6)	0.0138 (7)	0.0038 (5)	−0.0008 (5)	0.0022 (6)
C50	0.0215 (7)	0.0173 (7)	0.0216 (8)	0.0059 (6)	0.0031 (6)	0.0079 (6)
C34	0.0125 (6)	0.0182 (7)	0.0143 (7)	0.0034 (5)	0.0020 (5)	0.0031 (6)
C37	0.0192 (7)	0.0146 (6)	0.0150 (7)	0.0043 (5)	0.0022 (5)	0.0050 (6)
C58	0.0199 (7)	0.0205 (7)	0.0154 (7)	0.0053 (6)	−0.0010 (6)	0.0031 (6)
C31	0.0217 (7)	0.0153 (6)	0.0218 (8)	0.0048 (5)	0.0025 (6)	0.0100 (6)
C61	0.0243 (8)	0.0297 (8)	0.0248 (9)	0.0115 (7)	0.0003 (6)	0.0065 (7)
C54	0.0221 (7)	0.0170 (7)	0.0217 (8)	0.0050 (6)	0.0036 (6)	0.0062 (6)
C33	0.0113 (6)	0.0167 (6)	0.0153 (7)	0.0033 (5)	0.0030 (5)	0.0056 (6)
C56	0.0289 (8)	0.0264 (8)	0.0150 (8)	0.0059 (6)	−0.0005 (6)	−0.0002 (6)
C35	0.0210 (7)	0.0241 (7)	0.0125 (7)	0.0068 (6)	0.0009 (5)	0.0072 (6)
C60	0.0198 (7)	0.0350 (9)	0.0254 (8)	0.0055 (6)	0.0071 (6)	0.0104 (7)
C53	0.0325 (8)	0.0192 (7)	0.0247 (8)	0.0101 (6)	0.0118 (7)	0.0067 (6)
C52	0.0399 (9)	0.0148 (7)	0.0169 (8)	0.0050 (6)	0.0035 (7)	0.0026 (6)
C59	0.0245 (7)	0.0243 (8)	0.0231 (8)	0.0028 (6)	0.0023 (6)	0.0119 (7)
C55	0.0239 (8)	0.0291 (8)	0.0276 (9)	−0.0054 (7)	0.0026 (7)	0.0017 (7)
Cl4	0.01841 (17)	0.0384 (2)	0.0331 (2)	0.00178 (15)	0.00127 (15)	0.01215 (18)
Cl3	0.0389 (2)	0.0291 (2)	0.0496 (3)	0.01544 (17)	0.0146 (2)	0.0123 (2)
C63	0.0263 (8)	0.0249 (8)	0.0262 (8)	0.0083 (6)	0.0062 (6)	0.0149 (7)
C65	0.0296 (8)	0.0228 (8)	0.0301 (9)	0.0058 (6)	0.0090 (7)	0.0130 (7)
C64	0.0190 (7)	0.0243 (7)	0.0264 (8)	0.0035 (6)	0.0025 (6)	0.0146 (7)
C67	0.0168 (7)	0.0407 (10)	0.0444 (11)	0.0047 (7)	0.0024 (7)	0.0296 (9)
C68	0.0305 (8)	0.0249 (8)	0.0304 (9)	−0.0012 (7)	−0.0021 (7)	0.0138 (7)
C66	0.0270 (8)	0.0356 (9)	0.0481 (11)	0.0154 (7)	0.0151 (8)	0.0267 (9)

### Geometric parameters (Å, º)

**Table:**

S1—O1	1.4397 (11)	O7—C41	1.2168 (18)
S1—O2	1.4361 (10)	O8—C45	1.2146 (17)
S1—N1	1.6303 (12)	N5—C41	1.3991 (18)
S1—C1	1.7789 (14)	N5—C45	1.4049 (18)
Cl6—C70	1.7313 (16)	N5—C40	1.4677 (18)
Cl5—C69	1.7308 (16)	N4—C39	1.4694 (17)
O3—C13	1.2181 (18)	N4—C48	1.4853 (17)
O4—C17	1.2124 (17)	C47—H47	0.9500
N2—C13	1.3952 (18)	C47—C46	1.404 (2)
N2—C17	1.4065 (18)	C47—C42^ii^	1.3808 (19)
N2—C12	1.4706 (17)	C41—C42	1.478 (2)
N1—C11	1.4701 (17)	C38—C29	1.433 (2)
N1—C20	1.4834 (17)	C38—C37	1.4186 (19)
C13—C14	1.4812 (19)	C38—C33	1.4292 (19)
C1—C2	1.376 (2)	C44—C43	1.4139 (19)
C1—C10	1.432 (2)	C44—C46	1.380 (2)
C2—H2	0.9500	C44—C45	1.483 (2)
C2—C3	1.409 (2)	C49—C48	1.5117 (19)
N3—C6	1.4189 (19)	C49—C50	1.394 (2)
N3—C28	1.456 (2)	C49—C54	1.391 (2)
N3—C27	1.476 (2)	C32—H32	0.9500
C15—C15^i^	1.413 (3)	C32—C31	1.367 (2)
C15—C16	1.4118 (19)	C32—C33	1.4208 (19)
C15—C14	1.410 (2)	C29—C30	1.376 (2)
C18—H18	0.9500	N6—C34	1.4169 (19)
C18—C16	1.376 (2)	N6—C56	1.456 (2)
C18—C19	1.407 (2)	N6—C55	1.474 (2)
C4—H4	0.9500	C30—H30	0.9500
C4—C5	1.4190 (19)	C30—C31	1.411 (2)
C4—C3	1.367 (2)	C43—C43^ii^	1.416 (3)
C16—C17	1.4841 (19)	C43—C42	1.408 (2)
C23—H23	0.9500	C57—C62	1.388 (2)
C23—C22	1.395 (2)	C57—C58	1.391 (2)
C23—C24	1.384 (2)	C62—H62	0.9500
C19—H19	0.9500	C62—C61	1.386 (2)
C19—C14^i^	1.3787 (19)	C46—H46	0.9500
C12—H12A	0.9900	C42—C47^ii^	1.3808 (19)
C12—H12B	0.9900	C39—H39A	0.9900
C12—C11	1.522 (2)	C39—H39B	0.9900
C5—C10	1.4285 (19)	C39—C40	1.527 (2)
C5—C6	1.437 (2)	C51—H51	0.9500
C10—C9	1.4194 (19)	C51—C50	1.394 (2)
C21—C22	1.392 (2)	C51—C52	1.384 (2)
C21—C20	1.5134 (19)	C48—H48A	0.9900
C21—C26	1.393 (2)	C48—H48B	0.9900
C14—C19^i^	1.3787 (19)	C36—H36	0.9500
C3—H3	0.9500	C36—C37	1.368 (2)
C6—C7	1.374 (2)	C36—C35	1.410 (2)
C22—H22	0.9500	C40—H40A	0.9900
C9—H9	0.9500	C40—H40B	0.9900
C9—C8	1.365 (2)	C50—H50	0.9500
C11—H11A	0.9900	C34—C33	1.437 (2)
C11—H11B	0.9900	C34—C35	1.378 (2)
C25—H25	0.9500	C37—H37	0.9500
C25—C24	1.390 (2)	C58—C59	1.387 (2)
C25—C26	1.386 (2)	C31—H31	0.9500
C8—H8	0.9500	C61—H61	0.9500
C8—C7	1.412 (2)	C61—C60	1.384 (2)
C73—H73	0.9500	C54—H54	0.9500
C73—C74	1.382 (3)	C54—C53	1.388 (2)
C73—C72	1.380 (3)	C56—H56A	0.9800
C7—H7	0.9500	C56—H56B	0.9800
C74—H74	0.9500	C56—H56C	0.9800
C74—C69	1.391 (2)	C35—H35	0.9500
C69—C70	1.384 (2)	C60—H60	0.9500
C20—H20A	0.9900	C60—C59	1.384 (2)
C20—H20B	0.9900	C53—H53	0.9500
C70—C71	1.385 (2)	C53—C52	1.388 (2)
C24—H24	0.9500	C52—H52	0.9500
C28—H28A	0.9800	C59—H59	0.9500
C28—H28B	0.9800	C55—H55A	0.9800
C28—H28C	0.9800	C55—H55B	0.9800
C26—H26	0.9500	C55—H55C	0.9800
C71—H71	0.9500	Cl4—C64	1.7274 (15)
C71—C72	1.383 (3)	Cl3—C63	1.7278 (17)
C27—H27A	0.9800	C63—C64	1.388 (2)
C27—H27B	0.9800	C63—C68	1.390 (2)
C27—H27C	0.9800	C65—H65	0.9500
C72—H72	0.9500	C65—C64	1.388 (2)
S2—O5	1.4391 (11)	C65—C66	1.383 (2)
S2—O6	1.4347 (11)	C67—H67	0.9500
S2—N4	1.6323 (12)	C67—C68	1.361 (3)
S2—C29	1.7815 (14)	C67—C66	1.409 (3)
Cl1—C57	1.7341 (15)	C68—H68	0.9500
Cl2—C58	1.7293 (15)	C66—H66	0.9500
			
O1—S1—N1	109.76 (6)	C39—N4—S2	117.29 (9)
O1—S1—C1	106.07 (7)	C39—N4—C48	117.42 (11)
O2—S1—O1	118.32 (7)	C48—N4—S2	120.32 (9)
O2—S1—N1	107.61 (6)	C46—C47—H47	119.8
O2—S1—C1	108.46 (6)	C42^ii^—C47—H47	119.8
N1—S1—C1	105.96 (6)	C42^ii^—C47—C46	120.35 (13)
C13—N2—C17	125.20 (12)	O7—C41—N5	120.01 (13)
C13—N2—C12	116.53 (11)	O7—C41—C42	122.72 (13)
C17—N2—C12	118.27 (11)	N5—C41—C42	117.27 (12)
C11—N1—S1	117.28 (9)	C37—C38—C29	124.03 (13)
C11—N1—C20	117.11 (11)	C37—C38—C33	118.94 (13)
C20—N1—S1	121.14 (9)	C33—C38—C29	117.03 (12)
O3—C13—N2	120.21 (13)	C43—C44—C45	119.78 (12)
O3—C13—C14	122.57 (13)	C46—C44—C43	120.51 (13)
N2—C13—C14	117.22 (12)	C46—C44—C45	119.70 (12)
C2—C1—S1	116.75 (11)	C50—C49—C48	120.56 (13)
C2—C1—C10	121.69 (13)	C54—C49—C48	120.66 (13)
C10—C1—S1	121.45 (10)	C54—C49—C50	118.77 (14)
C1—C2—H2	119.9	C31—C32—H32	119.3
C1—C2—C3	120.12 (13)	C31—C32—C33	121.38 (13)
C3—C2—H2	119.9	C33—C32—H32	119.3
C6—N3—C28	115.76 (13)	C38—C29—S2	121.66 (10)
C6—N3—C27	113.39 (12)	C30—C29—S2	116.46 (11)
C28—N3—C27	109.56 (13)	C30—C29—C38	121.72 (13)
C16—C15—C15^i^	119.37 (16)	C34—N6—C56	115.73 (13)
C14—C15—C15^i^	119.12 (15)	C34—N6—C55	113.73 (12)
C14—C15—C16	121.51 (13)	C56—N6—C55	109.64 (13)
C16—C18—H18	120.0	C29—C30—H30	120.0
C16—C18—C19	120.10 (13)	C29—C30—C31	120.10 (13)
C19—C18—H18	120.0	C31—C30—H30	120.0
C5—C4—H4	119.2	C44—C43—C43^ii^	119.12 (16)
C3—C4—H4	119.2	C42—C43—C44	121.49 (13)
C3—C4—C5	121.53 (13)	C42—C43—C43^ii^	119.39 (16)
C15—C16—C17	119.85 (12)	C62—C57—Cl1	119.23 (12)
C18—C16—C15	120.56 (13)	C62—C57—C58	120.14 (14)
C18—C16—C17	119.59 (12)	C58—C57—Cl1	120.62 (12)
C22—C23—H23	119.8	C57—C62—H62	120.2
C24—C23—H23	119.8	C61—C62—C57	119.58 (15)
C24—C23—C22	120.31 (14)	C61—C62—H62	120.2
O4—C17—N2	121.06 (13)	C47—C46—H46	119.9
O4—C17—C16	122.35 (13)	C44—C46—C47	120.15 (13)
N2—C17—C16	116.59 (12)	C44—C46—H46	119.9
C18—C19—H19	119.9	O8—C45—N5	120.90 (13)
C14^i^—C19—C18	120.23 (13)	O8—C45—C44	122.48 (13)
C14^i^—C19—H19	119.9	N5—C45—C44	116.62 (12)
N2—C12—H12A	109.2	C47^ii^—C42—C41	119.85 (13)
N2—C12—H12B	109.2	C47^ii^—C42—C43	120.46 (13)
N2—C12—C11	112.25 (11)	C43—C42—C41	119.68 (12)
H12A—C12—H12B	107.9	N4—C39—H39A	109.1
C11—C12—H12A	109.2	N4—C39—H39B	109.1
C11—C12—H12B	109.2	N4—C39—C40	112.29 (11)
C4—C5—C10	119.27 (13)	H39A—C39—H39B	107.9
C4—C5—C6	121.13 (13)	C40—C39—H39A	109.1
C10—C5—C6	119.57 (13)	C40—C39—H39B	109.1
C5—C10—C1	117.12 (12)	C50—C51—H51	120.0
C9—C10—C1	123.99 (13)	C52—C51—H51	119.9
C9—C10—C5	118.88 (13)	C52—C51—C50	120.10 (15)
C22—C21—C20	120.26 (13)	N4—C48—C49	114.11 (11)
C22—C21—C26	118.75 (13)	N4—C48—H48A	108.7
C26—C21—C20	120.98 (13)	N4—C48—H48B	108.7
C15—C14—C13	119.63 (12)	C49—C48—H48A	108.7
C19^i^—C14—C13	119.76 (13)	C49—C48—H48B	108.7
C19^i^—C14—C15	120.61 (13)	H48A—C48—H48B	107.6
C2—C3—H3	120.1	C37—C36—H36	119.3
C4—C3—C2	119.80 (13)	C37—C36—C35	121.49 (13)
C4—C3—H3	120.1	C35—C36—H36	119.3
N3—C6—C5	117.79 (13)	N5—C40—C39	111.48 (11)
C7—C6—N3	123.13 (13)	N5—C40—H40A	109.3
C7—C6—C5	119.05 (13)	N5—C40—H40B	109.3
C23—C22—H22	119.8	C39—C40—H40A	109.3
C21—C22—C23	120.39 (14)	C39—C40—H40B	109.3
C21—C22—H22	119.8	H40A—C40—H40B	108.0
C10—C9—H9	120.0	C49—C50—H50	119.8
C8—C9—C10	119.91 (13)	C51—C50—C49	120.50 (14)
C8—C9—H9	120.0	C51—C50—H50	119.8
N1—C11—C12	112.44 (11)	N6—C34—C33	118.05 (13)
N1—C11—H11A	109.1	C35—C34—N6	122.96 (13)
N1—C11—H11B	109.1	C35—C34—C33	118.94 (13)
C12—C11—H11A	109.1	C38—C37—H37	120.0
C12—C11—H11B	109.1	C36—C37—C38	119.94 (13)
H11A—C11—H11B	107.8	C36—C37—H37	120.0
C24—C25—H25	120.0	C57—C58—Cl2	121.08 (12)
C26—C25—H25	120.0	C59—C58—Cl2	119.07 (12)
C26—C25—C24	120.04 (15)	C59—C58—C57	119.85 (14)
C9—C8—H8	119.2	C32—C31—C30	119.85 (13)
C9—C8—C7	121.58 (13)	C32—C31—H31	120.1
C7—C8—H8	119.2	C30—C31—H31	120.1
C74—C73—H73	119.8	C62—C61—H61	119.8
C72—C73—H73	119.8	C60—C61—C62	120.36 (15)
C72—C73—C74	120.47 (16)	C60—C61—H61	119.8
C6—C7—C8	120.58 (14)	C49—C54—H54	119.6
C6—C7—H7	119.7	C53—C54—C49	120.74 (14)
C8—C7—H7	119.7	C53—C54—H54	119.6
C73—C74—H74	120.2	C38—C33—C34	119.53 (13)
C73—C74—C69	119.51 (16)	C32—C33—C38	119.41 (13)
C69—C74—H74	120.2	C32—C33—C34	121.04 (13)
C74—C69—Cl5	119.49 (13)	N6—C56—H56A	109.5
C70—C69—Cl5	120.55 (13)	N6—C56—H56B	109.5
C70—C69—C74	119.96 (15)	N6—C56—H56C	109.5
N1—C20—C21	113.67 (11)	H56A—C56—H56B	109.5
N1—C20—H20A	108.8	H56A—C56—H56C	109.5
N1—C20—H20B	108.8	H56B—C56—H56C	109.5
C21—C20—H20A	108.8	C36—C35—H35	119.7
C21—C20—H20B	108.8	C34—C35—C36	120.66 (13)
H20A—C20—H20B	107.7	C34—C35—H35	119.7
C69—C70—Cl6	120.85 (13)	C61—C60—H60	120.0
C69—C70—C71	120.14 (15)	C59—C60—C61	120.07 (15)
C71—C70—Cl6	119.01 (13)	C59—C60—H60	120.0
C23—C24—C25	119.59 (14)	C54—C53—H53	119.9
C23—C24—H24	120.2	C52—C53—C54	120.14 (15)
C25—C24—H24	120.2	C52—C53—H53	119.9
N3—C28—H28A	109.5	C51—C52—C53	119.75 (15)
N3—C28—H28B	109.5	C51—C52—H52	120.1
N3—C28—H28C	109.5	C53—C52—H52	120.1
H28A—C28—H28B	109.5	C58—C59—H59	120.0
H28A—C28—H28C	109.5	C60—C59—C58	119.98 (15)
H28B—C28—H28C	109.5	C60—C59—H59	120.0
C21—C26—H26	119.6	N6—C55—H55A	109.5
C25—C26—C21	120.89 (14)	N6—C55—H55B	109.5
C25—C26—H26	119.6	N6—C55—H55C	109.5
C70—C71—H71	120.1	H55A—C55—H55B	109.5
C72—C71—C70	119.83 (16)	H55A—C55—H55C	109.5
C72—C71—H71	120.1	H55B—C55—H55C	109.5
N3—C27—H27A	109.5	C64—C63—Cl3	120.99 (12)
N3—C27—H27B	109.5	C64—C63—C68	119.41 (15)
N3—C27—H27C	109.5	C68—C63—Cl3	119.61 (13)
H27A—C27—H27B	109.5	C64—C65—H65	120.2
H27A—C27—H27C	109.5	C66—C65—H65	120.2
H27B—C27—H27C	109.5	C66—C65—C64	119.70 (16)
C73—C72—C71	120.08 (16)	C63—C64—Cl4	121.09 (12)
C73—C72—H72	120.0	C65—C64—Cl4	118.60 (13)
C71—C72—H72	120.0	C65—C64—C63	120.31 (14)
O5—S2—N4	109.36 (6)	C68—C67—H67	120.2
O5—S2—C29	105.88 (7)	C68—C67—C66	119.59 (15)
O6—S2—O5	118.74 (7)	C66—C67—H67	120.2
O6—S2—N4	107.79 (6)	C63—C68—H68	119.5
O6—S2—C29	108.16 (6)	C67—C68—C63	121.05 (16)
N4—S2—C29	106.27 (6)	C67—C68—H68	119.5
C41—N5—C45	125.05 (12)	C65—C66—C67	119.94 (16)
C41—N5—C40	116.49 (12)	C65—C66—H66	120.0
C45—N5—C40	118.46 (11)	C67—C66—H66	120.0
			
S1—N1—C11—C12	−132.54 (10)	Cl2—C58—C59—C60	179.14 (13)
S1—N1—C20—C21	−94.83 (13)	O7—C41—C42—C47^ii^	1.3 (2)
S1—C1—C2—C3	−177.24 (11)	O7—C41—C42—C43	−177.36 (13)
S1—C1—C10—C5	171.10 (10)	O5—S2—N4—C39	−29.11 (12)
S1—C1—C10—C9	−9.81 (19)	O5—S2—N4—C48	125.32 (11)
Cl6—C70—C71—C72	−179.28 (14)	O5—S2—C29—C38	−165.99 (11)
Cl5—C69—C70—Cl6	−1.77 (19)	O5—S2—C29—C30	9.43 (13)
Cl5—C69—C70—C71	178.36 (13)	O6—S2—N4—C39	−159.49 (10)
O3—C13—C14—C15	179.55 (13)	O6—S2—N4—C48	−5.06 (12)
O3—C13—C14—C19^i^	−0.6 (2)	O6—S2—C29—C38	−37.72 (13)
O1—S1—N1—C11	−32.20 (12)	O6—S2—C29—C30	137.70 (11)
O1—S1—N1—C20	123.41 (11)	N5—C41—C42—C47^ii^	−178.82 (13)
O1—S1—C1—C2	8.55 (13)	N5—C41—C42—C43	2.53 (19)
O1—S1—C1—C10	−167.82 (11)	N4—S2—C29—C38	77.78 (12)
O2—S1—N1—C11	−162.21 (10)	N4—S2—C29—C30	−106.80 (11)
O2—S1—N1—C20	−6.60 (12)	N4—C39—C40—N5	61.25 (15)
O2—S1—C1—C2	136.63 (11)	C41—N5—C45—O8	177.01 (13)
O2—S1—C1—C10	−39.74 (13)	C41—N5—C45—C44	−2.55 (19)
N2—C13—C14—C15	0.36 (19)	C41—N5—C40—C39	77.33 (15)
N2—C13—C14—C19^i^	−179.77 (13)	C38—C29—C30—C31	−1.2 (2)
N2—C12—C11—N1	61.88 (15)	C44—C43—C42—C47^ii^	−179.98 (13)
N1—S1—C1—C2	−108.08 (11)	C44—C43—C42—C41	−1.3 (2)
N1—S1—C1—C10	75.55 (12)	C49—C54—C53—C52	0.9 (2)
C13—N2—C17—O4	−179.49 (13)	C29—S2—N4—C39	84.76 (11)
C13—N2—C17—C16	1.17 (19)	C29—S2—N4—C48	−120.81 (11)
C13—N2—C12—C11	77.16 (15)	C29—C38—C37—C36	179.38 (13)
C1—S1—N1—C11	81.92 (11)	C29—C38—C33—C32	7.81 (19)
C1—S1—N1—C20	−122.47 (11)	C29—C38—C33—C34	−173.81 (12)
C1—C2—C3—C4	4.5 (2)	C29—C30—C31—C32	4.9 (2)
C1—C10—C9—C8	179.50 (13)	N6—C34—C33—C38	174.87 (12)
C2—C1—C10—C5	−5.09 (19)	N6—C34—C33—C32	−6.8 (2)
C2—C1—C10—C9	173.99 (13)	N6—C34—C35—C36	−179.89 (13)
N3—C6—C7—C8	−179.25 (13)	C43—C44—C46—C47	1.3 (2)
C15^i^—C15—C16—C18	0.4 (2)	C43—C44—C45—O8	−175.86 (13)
C15^i^—C15—C16—C17	−179.83 (14)	C43—C44—C45—N5	3.70 (18)
C15^i^—C15—C14—C13	179.77 (15)	C43^ii^—C43—C42—C47^ii^	−0.8 (2)
C15^i^—C15—C14—C19^i^	−0.1 (2)	C43^ii^—C43—C42—C41	177.83 (15)
C15—C16—C17—O4	−179.57 (13)	C57—C62—C61—C60	−0.8 (2)
C15—C16—C17—N2	−0.24 (18)	C57—C58—C59—C60	−0.2 (2)
C18—C16—C17—O4	0.2 (2)	C62—C57—C58—Cl2	179.84 (12)
C18—C16—C17—N2	179.56 (12)	C62—C57—C58—C59	−0.8 (2)
C4—C5—C10—C1	7.51 (19)	C62—C61—C60—C59	−0.3 (3)
C4—C5—C10—C9	−171.62 (13)	C46—C44—C43—C43^ii^	−0.2 (2)
C4—C5—C6—N3	−7.2 (2)	C46—C44—C43—C42	178.94 (13)
C4—C5—C6—C7	170.94 (13)	C46—C44—C45—O8	3.4 (2)
C16—C15—C14—C13	0.5 (2)	C46—C44—C45—N5	−177.06 (12)
C16—C15—C14—C19^i^	−179.38 (13)	C45—N5—C41—O7	179.39 (13)
C16—C18—C19—C14^i^	−1.2 (2)	C45—N5—C41—C42	−0.5 (2)
C17—N2—C13—O3	179.57 (13)	C45—N5—C40—C39	−102.07 (14)
C17—N2—C13—C14	−1.2 (2)	C45—C44—C43—C43^ii^	179.00 (14)
C17—N2—C12—C11	−103.74 (14)	C45—C44—C43—C42	−1.8 (2)
C19—C18—C16—C15	0.6 (2)	C45—C44—C46—C47	−177.95 (13)
C19—C18—C16—C17	−179.24 (13)	C42^ii^—C47—C46—C44	−1.3 (2)
C12—N2—C13—O3	−1.41 (19)	C39—N4—C48—C49	61.96 (16)
C12—N2—C13—C14	177.80 (12)	C48—N4—C39—C40	71.72 (15)
C12—N2—C17—O4	1.49 (19)	C48—C49—C50—C51	−179.45 (13)
C12—N2—C17—C16	−177.84 (11)	C48—C49—C54—C53	178.52 (13)
C5—C4—C3—C2	−1.9 (2)	C40—N5—C41—O7	0.03 (19)
C5—C10—C9—C8	−1.4 (2)	C40—N5—C41—C42	−179.86 (12)
C5—C6—C7—C8	2.7 (2)	C40—N5—C45—O8	−3.64 (19)
C10—C1—C2—C3	−0.9 (2)	C40—N5—C45—C44	176.80 (11)
C10—C5—C6—N3	174.66 (12)	C50—C49—C48—N4	−93.61 (16)
C10—C5—C6—C7	−7.2 (2)	C50—C49—C54—C53	−0.2 (2)
C10—C9—C8—C7	−3.2 (2)	C50—C51—C52—C53	−0.3 (2)
C14—C15—C16—C18	179.66 (13)	C37—C38—C29—S2	−10.89 (19)
C14—C15—C16—C17	−0.54 (19)	C37—C38—C29—C30	173.93 (14)
C3—C4—C5—C10	−4.2 (2)	C37—C38—C33—C32	−171.33 (13)
C3—C4—C5—C6	177.62 (13)	C37—C38—C33—C34	7.05 (19)
C6—C5—C10—C1	−174.30 (12)	C37—C36—C35—C34	2.9 (2)
C6—C5—C10—C9	6.57 (19)	C58—C57—C62—C61	1.3 (2)
C22—C23—C24—C25	−0.7 (2)	C31—C32—C33—C38	−4.4 (2)
C22—C21—C20—N1	−105.22 (15)	C31—C32—C33—C34	177.27 (13)
C22—C21—C26—C25	−0.9 (2)	C61—C60—C59—C58	0.8 (2)
C9—C8—C7—C6	2.5 (2)	C54—C49—C48—N4	87.67 (16)
C11—N1—C20—C21	60.82 (16)	C54—C49—C50—C51	−0.7 (2)
C73—C74—C69—Cl5	−178.72 (13)	C54—C53—C52—C51	−0.7 (2)
C73—C74—C69—C70	0.8 (3)	C33—C38—C29—S2	170.02 (10)
C74—C73—C72—C71	−0.7 (3)	C33—C38—C29—C30	−5.2 (2)
C74—C69—C70—Cl6	178.69 (13)	C33—C38—C37—C36	−1.5 (2)
C74—C69—C70—C71	−1.2 (2)	C33—C32—C31—C30	−2.1 (2)
C69—C70—C71—C72	0.6 (3)	C33—C34—C35—C36	2.7 (2)
C20—N1—C11—C12	70.86 (15)	C56—N6—C34—C33	161.71 (13)
C20—C21—C22—C23	−179.45 (13)	C56—N6—C34—C35	−15.7 (2)
C20—C21—C26—C25	178.09 (13)	C35—C36—C37—C38	−3.5 (2)
C70—C71—C72—C73	0.4 (3)	C35—C34—C33—C38	−7.6 (2)
C24—C23—C22—C21	1.3 (2)	C35—C34—C33—C32	170.71 (13)
C24—C25—C26—C21	1.5 (2)	C52—C51—C50—C49	1.0 (2)
C28—N3—C6—C5	161.65 (13)	C55—N6—C34—C33	−70.03 (17)
C28—N3—C6—C7	−16.4 (2)	C55—N6—C34—C35	112.59 (17)
C26—C21—C22—C23	−0.5 (2)	Cl3—C63—C64—Cl4	0.69 (19)
C26—C21—C20—N1	75.81 (16)	Cl3—C63—C64—C65	−179.71 (13)
C26—C25—C24—C23	−0.7 (2)	Cl3—C63—C68—C67	179.29 (13)
C27—N3—C6—C5	−70.49 (17)	C64—C63—C68—C67	−1.2 (2)
C27—N3—C6—C7	111.47 (17)	C64—C65—C66—C67	−0.9 (3)
C72—C73—C74—C69	0.1 (3)	C68—C63—C64—Cl4	−178.84 (12)
S2—N4—C39—C40	−133.10 (10)	C68—C63—C64—C65	0.8 (2)
S2—N4—C48—C49	−92.44 (13)	C68—C67—C66—C65	0.5 (3)
S2—C29—C30—C31	−176.58 (11)	C66—C65—C64—Cl4	179.88 (13)
Cl1—C57—C62—C61	−177.56 (12)	C66—C65—C64—C63	0.3 (2)
Cl1—C57—C58—Cl2	−1.31 (18)	C66—C67—C68—C63	0.5 (3)
Cl1—C57—C58—C59	178.02 (12)		

Symmetry codes: (i) −*x*, −*y*+2, −*z*+1; (ii) −*x*, −*y*+2, −*z*+2.

### Hydrogen-bond geometry (Å, º)

Cg, Cg5', Cg6', Cg7' and Cg8' are the centroids of atoms C63–C68, C21–C26, C49–C54, C1–C10 and C29–C38, respectively.

**Table:**

*D*—H···*A*	*D*—H	H···*A*	*D*···*A*	*D*—H···*A*
C8—H8···O5	0.95	2.53	3.1926 (19)	127
C19—H19···O7	0.95	2.55	3.2615 (19)	132
C31—H31···O3^i^	0.95	2.59	3.3818 (19)	141
C47—H47···O3^iii^	0.95	2.58	3.2148 (19)	125
C60—H60···O2	0.95	2.54	3.335 (2)	142
C68—H68···Cl1^iii^	0.95	2.79	3.5922 (19)	142
C72—H72···O6	0.95	2.50	3.293 (2)	141
C71—H71···*Cg*	0.95	2.99	3.813 (2)	145
C55—H55*A*···*Cg*8′^iv^	0.98	2.94	3.632 (2)	129
C27—H27*A*···*Cg*7′^v^	0.98	3.03	3.585 (2)	117
C20—H20*B*···*Cg*6′^vi^	0.99	3.12	3.6054 (17)	111
C48—H48*B*···*Cg*5′^vi^	0.99	3.13	3.6180 (17)	112

Symmetry codes: (i) −*x*, −*y*+2, −*z*+1; (iii) *x*, *y*, *z*+1; (iv) −*x*+1, −*y*+2, −*z*+2; (v) −*x*+1, −*y*+2, −*z*+1; (vi) −*x*, −*y*+1, −*z*+1.
